# Hematopoietic Wnts Modulate Endochondral Ossification During Fracture Healing

**DOI:** 10.3389/fendo.2021.667480

**Published:** 2021-05-24

**Authors:** Kenon Chua, Victor K. Lee, Cheri Chan, Andy Yew, Eric Yeo, David M. Virshup

**Affiliations:** ^1^ Programme in Cancer and Stem Cell Biology, Duke-NUS Medical School, Singapore, Singapore; ^2^ Department of Orthopedic Surgery, Singapore General Hospital, Singapore, Singapore; ^3^ Programme in Musculoskeletal Sciences Academic Clinical Program, SingHealth/Duke-NUS, Singapore, Singapore; ^4^ Department of Pathology, National University of Singapore, Singapore, Singapore; ^5^ Department of Pediatrics, Duke University, Durham, NC, United States

**Keywords:** fracture healing, bone formation, Wnt signaling, osteoclast, hematopoietic Wnts

## Abstract

Wnt signaling plays a critical role in bone formation, homeostasis, and injury repair. Multiple cell types in bone have been proposed to produce the Wnts required for these processes. The specific role of Wnts produced from cells of hematopoietic origin has not been previously characterized. Here, we examined if hematopoietic Wnts play a role in physiological musculoskeletal development and in fracture healing. Wnt secretion from hematopoietic cells was blocked by genetic knockout of the essential Wnt modifying enzyme PORCN, achieved by crossing *Vav-Cre* transgenic mice with *Porcn^flox^* mice. Knockout mice were compared with their wild-type littermates for musculoskeletal development including bone quantity and quality at maturation. Fracture healing including callus quality and quantity was assessed in a diaphyseal fracture model using quantitative micro computer-assisted tomographic scans, histological analysis, as well as biomechanical torsional and 4-point bending stress tests. The hematopoietic *Porcn* knockout mice had normal musculoskeletal development, with normal bone quantity and quality on micro-CT scans of the vertebrae. They also had normal gross skeletal dimensions and normal bone strength. Hematopoietic Wnt depletion in the healing fracture resulted in fewer osteoclasts in the fracture callus, with a resultant delay in callus remodeling. All calluses eventually progressed to full maturation. Hematopoietic Wnts, while not essential, modulate osteoclast numbers during fracture healing. These osteoclasts participate in callus maturation and remodeling. This demonstrates the importance of diverse Wnt sources in bone repair.

## Highlights

- Hematopoietic Wnts are dispensable for normal skeletal development, growth and maturation.
- Bone mass accrual and bone quality are not adversely affected by hematopoietic Wnt depletion.
- Depletion of hematopoietic Wnts results in fewer osteoclasts and delayed maturation of the fracture callus after bone injury.
- Hematopoietic Wnts are dispensable for completion of fracture healing.

## Introduction

Bone is a complex tissue that is structurally important for force transmission and locomotion, as well as mineral metabolism and hematopoiesis. The Wnt signaling pathway is a key modulator of bone formation (1–4). Wnts influence prenatal skeletal development, as well as post-partum skeletal growth and maturation. Wnts are also critically important in bone mass accrual and adult bone hemostasis in the skeletally mature individual.

There are 19 distinct Wnt ligands in the human genome that function *via* short range cell to cell signaling (5–8). Different Wnts can function in different processes, broadly categorized into β-catenin dependent and independent pathways, both of which are involved in bone formation at different time points during development and fracture healing. Early in bone development, Wnt/β-catenin signaling through Frizzled and LRP5/6 receptors inhibits chondrogenesis. Conversely, WNT5A interacts with its receptor ROR2 to antagonize the Wnt/β-catenin pathway to induce local chondrogenesis by stimulating cartilage nodule formation (9, 10). Wnt/β-catenin signaling, in contrast, promotes osteoblastic differentiation (11) and mineralization (12). This is important in late phase fracture callus maturation as well as bone growth. Besides maintaining the osteoblasts, Wnts also enhance proliferation and prevent differentiation of the osteoclast precursor cells, regulating the number of mature osteoclasts that are critical for callus remodeling. Wnt signaling is thus extremely important for multiple aspects of fracture healing (13–16).

The Wnts regulating bone formation and repair can be produced by multiple cell types, the most well-known being osteoblasts (11). Another potential source are the cells of hematopoietic origin (hereafter called hematopoietic Wnts). Hematopoietic stem cells differentiate and proliferate into various blood components every day. Bone plays a role in regulating hematopoiesis (17). Conversely, cells of hematopoietic origin, specifically monocytes and tissue macrophages, are important in injury repair (18–20). Macrophages migrate to sites of tissue injury and produce Wnts as well as multiple cytokines and other factors to aid in tissue repair (21, 22). Whether hematopoietic and macrophage derived Wnts also contribute to fracture healing and bone formation is not known.

Fracture non-unions and delayed unions are common clinical problems worldwide, with up to 18.5 percent incidence in tibia diaphyseal fractures reported (23). Non-union is defined clinically as the arrest of progression to union at the fracture site with persistent pain and mobility for six months or more. The causes of non-union are categorized into two broad groups. The first is due to mechanical factors that impair fracture healing, such as poor strain environment or excessive motion. The second is caused by biological factors. This includes a multitude of causes that have a cumulative or additive effects, including local bone marrow suppression, poor vascularity, immunosuppression, and aberrant cellular signaling (24–27). Introducing autologous bone graft and bone marrow aspirate concentrate to the fracture site to promote healing in fracture non-unions has proven to be clinically efficacious (28–31). These therapeutic interventions introduce several cell types, including cells of hematopoietic origin that are important for fracture healing (19). Hematopoietic cells are also known to express multiple Wnt ligands, but their function is controversial. Kabiri et al. made the unexpected and surprising discovery, examining mice with genetic knockout of *Porcn* in cells of hematopoietic lineage using three (Vav, Mx1, and Rosa26) distinct Cre drivers, that intrinsic Wnt production was not required for the stemness, regeneration, nor differentiation of the hematopoietic compartment (32). Why then do hematopoietic cells make Wnts? Since we know that Wnts function as short-range signaling molecules, we hypothesized that Wnts secreted from cells of hematopoietic origin (hereafter designated as hematopoietic Wnts) at the site of injury might play an additive role in modulating bone formation.

**Figure 1:**
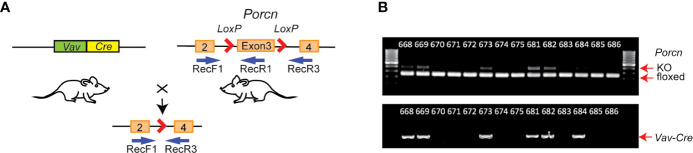
Generation of hematopoietic Wnt-depleted mice. **(A)** *Porcn^flox^* mice were crossed with *Vav-Cre* mice, resulting in *Vav-Cre/Porcn^flox^* mice with excisional deletion of *Porcn* exon 3 in hematopoietic and other tissues expressing *Vav-Cre*. Relative annealing positions of complementary primers used for PCR from mouse genomic DNA is shown (RecF1, RecR1, and RecR3). **(B)** PCR genotyping using DNA from tail clippings. Each lane is from an individual mouse. Upper panel, *Vav-Cre/Porcn^flox^* mice samples exhibited 2 bands, a faster 128 bp band from RecF1/RecR1 (floxed), and a slower 248 bp band from RecF1/RecR3 (KO, knockout), since the tail clippings contain both hematopoietic and non-hematopoietic tissue. *Porcn^flox^* mice in the absence of *Vav-Cre* exhibit only a single PCR product (RecF1/RecR1).

**Figure 2:**
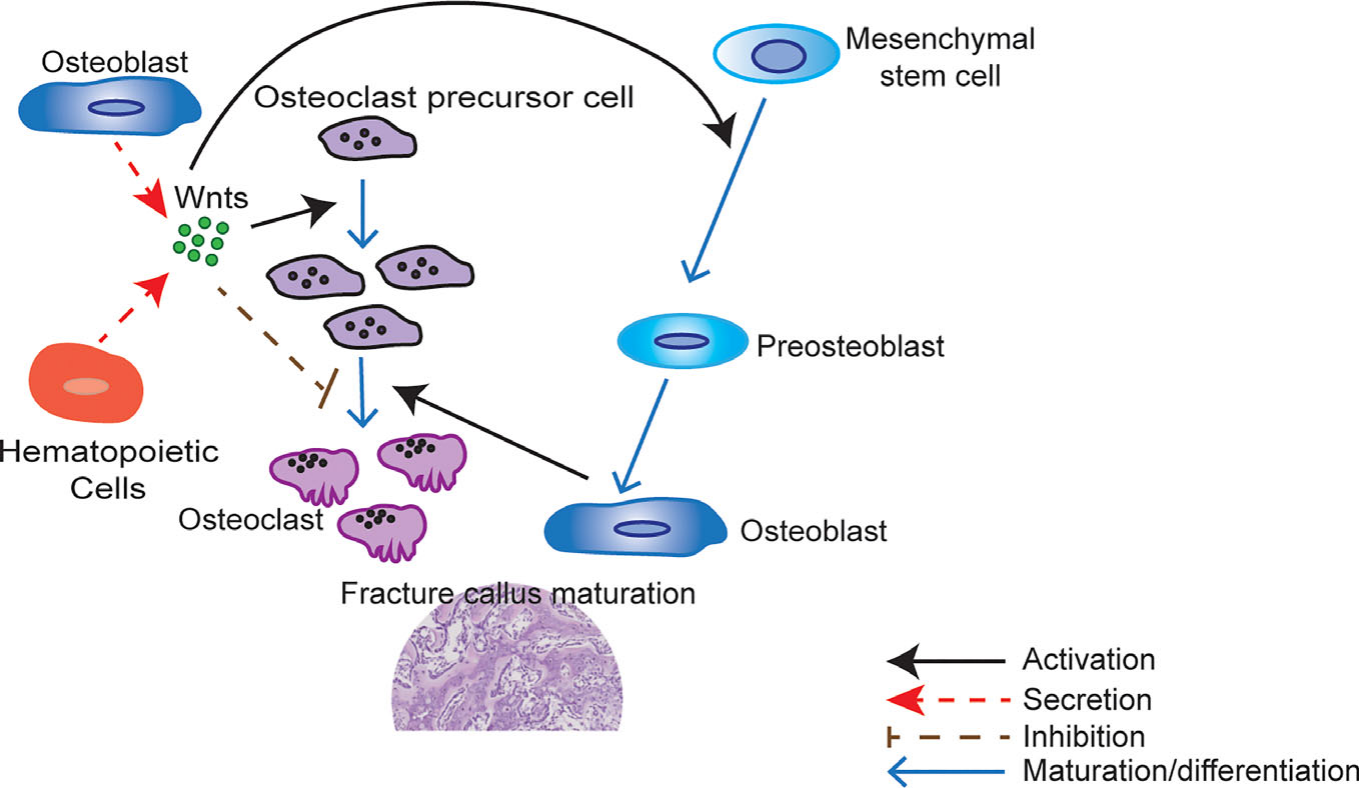
Wnts modulate fracture callus maturation. Hematopoietic and other cells within the bone microenvironment secrete Wnts. Wnt upregulation activates osteoclastic precursor cell proliferation but inhibits osteoclastic precursor cell maturation to osteoclast. Carefully regulated Wnt activity is therefore required for the correct number of mature osteoclasts to develop during fracture healing. Wnts are also responsible for the activation of mesenchymal stem cell differentiation to osteoblasts. These mature osteoblasts are responsible for the formation of woven bone. Osteoclasts also play a key role in callus maturation by resorption of cartilage during callus remodeling. A quantitative shortfall in the secreted Wnts may therefore result in a less mature callus with more cartilage and less woven bone by inhibiting the proliferation of osteoclast precursor cells.

To test the importance of hematopoietic Wnts in bone growth and fracture healing, we utilized the previously reported *Vav-Cre* x *Porcn^flox^* mouse model (**Figure 1**), where Wnt secretion is blocked specifically in cells of hematopoietic lineage (32). This is accomplished by knockout of the *Porcn* gene encoding an endoplasmic reticulum-resident membrane bound O-acyltransferase that responsible for palmitoleation of all Wnt molecules. This essential post-translational modification is required for the interaction with the Wnt transporter molecule WLS, which transports Wnts to the cell surface for secretion. Knockout of *Porcn* therefore results in an upstream inhibition of Wnt signaling, by inhibiting secretion of all Wnts (33, 34). We assessed skeletal development, growth and maturation, as well as fracture healing, in the absence of hematopoietic Wnts. We found that hematopoietic Wnts did indeed contribute quantitatively to fracture callus maturation. Hematopoietic Wnt depleted mice had fewer mature osteoclasts as well as more residual mineralized cartilage in the callus (**Figure 2**). We also observed a marginal decrease in skeletal length in the hematopoietic Wnt depleted mice. However, depletion of hematopoietic Wnts did not result in severe musculoskeletal abnormalities. This suggests that there is a physiological redundancy of secreted Wnts from other cell types for modulation of bone formation during development. Hematopoietic Wnts do play a role in modulating osteoclasts during fracture healing (**Figure 2**).

## Methods

### Mice Strains

Mice with a *Porcn* conditional null allele (*Porcn^flox^)* were generated as described (35). The *Porcn^flox^* mice were crossed with *Vav-Cre* mice [9, 14] to generate *Vav-Cre/Porcn^Del^* mice, with hematopoietic tissue-specific block in Wnt secretion (**Figure 1**). These mice were age and gender matched with their wild-type litter mates for comparison. Male mice and female mice were analyzed separately. Genotyping primer sequences are provided in the table **PRIMERS**. Animal housing, breeding and procedures were done in accordance with IACUC guidelines.

**Primers:** Genotyping primer sequences.

Primer	Sequence
*PORCN* Forward	CTGTTAAACCAAGACATGACCTTCA
*PORCN* Reverse 1	TAACTAGGACGCTTTGGGATAGGAT
*PORCN* Reverse 3	GTTCTGCCTTCCTAACCCATATAAC
*Vav* Forward	GGACATGTTCAGGGACAGGCA
*Vav* Reverse	CTCTGATTCTGGCAATTTCGGC

### Measurement of Developmental Weight and Dimensions

Mice pups were weaned at 3 weeks old and weighed at weekly intervals until past skeletal maturity (100 days old). Measurements of mouse bone length (nose to pelvis), right femur length and cranial size were done at skeletal maturity using digital calipers, after dissection of soft tissue. Both male and female mice were measured and analyzed. To facilitate readability and avoid figure redundancy, all figures henceforth are of female mice, since there were no gender specific differences in the results.

### Whole-Mount Skeletal Staining

Mice were sacrificed at 3 weeks of age and stained as described by Rigueur and Lyons (36). Soft tissue was removed, including the skin, fat, muscle and viscera, and the specimens were fixed in ethanol (95%). The whole mouse specimens were then sequentially stained with Alcian Blue and Alizarin Red stains.

### Fracture Surgery

Tibia diaphyseal fracture model was performed as described (19) at ~100 days of age. A 1 cm incision was made centered on the proximal tibia. The patella was identified. A stainless-steel pin (Entochrysis Stainless Mounting Insect Pin, Size 00) was inserted *via* the tibia plateau to stabilize the subsequent tibia fracture, and a diaphyseal osteotomy was then performed. Care was taken to ensure that the localization of the osteotomy was consistent from mouse to mouse, by referencing to the tibia tubercle. Routine analgesia was given perioperatively and the mice were allowed to weight bear as tolerated upon recovery. The mice were sacrificed at 2 weeks, 3 weeks or 4 weeks after the osteotomy was performed for callus analysis. The pin was removed from the tibia specimens after retrieval. Both male and female mice were analyzed separately at the 2-week, 3-week and 4-week time points after injury to exclude gender bias.

### Micro-Computed Tomography (Micro-CT, µCT)

Harvested specimens were dissected of soft tissue and scanned using high resolution micro-CT (Skyscan 1176, Bruker Micro-CT, Kontich, Belgium) at 9 µm resolution. Image acquisition was performed at 50 kV, 800 µA and 0.25 mm aluminum filter. Hydroxyapatite phantoms were scanned and used for bone mineral density (BMD) calibration.

Acquired images were reconstructed with NRecon and analyzed with CTAn (version 1.5.0, Skyscan, Bruker Micro-CT). For analysis of the cancellous bone, the region of interest encompassing the entire vertebrae body was manually defined to include the trabeculae bone only. Cortical bone was segmented out. This was used to generate a three-dimensional (3D) model CTvol (Skyscan Bruker Micro-CT) for analysis. For analysis of the tibial cortical bone, the midpoint of the tibia was used, and a 100-slice volume centered on this midpoint was defined as the region of interest. For analysis of the fracture callus, the midpoint of the callus was used to define a 3mm region of interest. The cortical bone from the fracture fragments within the callus was segmented out, leaving only the newly formed callus for analysis. The fibular callus was not included in our analysis.

### Histology

Fracture calluses were decalcified in Osteosoft (Merck) for 5 days and paraffin embedded after μCT scanning of bone. Serial sections of 3 μm were deparaffinized and rehydrated to water for hematoxylin and eosin (H&E), toluidine blue and Von Kossa Stain.

For hematoxylin and eosin (H&E) staining, tissue sections were stained in Shandon hematoxylin solution, differentiated in 1% acid alcohol, and immersed in ammonia with washing between steps. The sections were subsequently rinsed in 95% alcohol, counterstained in eosin-phloxine solution and rinsed before mounting.

Toluidine blue stain was used to identify the cartilaginous components of the callus. Sections were stained with 1% toluidine blue before being dehydrated and mounted. After staining, cartilage appears blue to purple, nuclei dark blue and all other tissue green.

Von Kossa staining was employed to monitor the mineralization of bone. After the sections were degreased and rehydrated, 2% silver nitrate solution was applied to each section, and the slides were exposed to strong light for 30 minutes. After the silver nitrate was removed, 5% sodium thiosulfate was added to the section for prior to rinsing with distilled water. The sections were then incubated with van Gieson working solution before mounting. After von Kossa staining, the mineralized bone appeared black, and less mineralized bone appearing pink.

Tartrate resistant acid phosphatase (TRAP) staining was used to stain osteoclasts. Demineralized samples were incubated in freshly made TRAP staining solution at 37°C for 30 minutes. The slides were then rinsed with distilled water and counterstained with 0.02% Fast Green for 30 seconds and then rinsed again with distilled water. Osteoclasts were stained red violet and the callus matrix was stained green.

### Histomorphometry Analysis

Histology cross-sections were referenced to the corresponding micro-CT images to identify those which represented the center of the fracture callus, and mid-section of the vertebrae. These sections were digitized and analyzed using the BioQuant Osteo software package (https://osteo.bioquant.com, Nashville, TN, USA). Total cartilage volume, total tissue volume, total trabecular bone volume, total trabecular bone surface area, trabecular diameter, trabecular number, trabecular spacing, fibrosis volume, total osteoblast surface, total osteoblast number, and osteoblast number per bone surface were analyzed. The results for osteoblast number, osteoblast number per bone surface, cartilage volume, and cartilage to total volume ratio were verified manually by a trained clinical pathologist (VKL) reviewing the digitized images of the calluses.

### Biomechanical Evaluation

Whole tibia fracture callus specimens were allocated for mechanical testing. All specimens were measured with a digital Vernier caliper (Mitutoyo Absolute, Mitutoyo, Japan), and the mid-point for the callus was determined. Intramedullary pins were removed prior to testing. A distance of 1.5 mm from the mid-point of the callus was marked superiorly and inferiorly. The 1.5 mm marking was used as a baseline to ensure that the tibia was centered, with equidistance from the mid-point of the callus. Superior and inferior ends of the tibia were embedded into stainless steel nuts using acrylic dental cement. Tibial specimens were wrapped in gauze soaked in saline solution to prevent the bone from dehydration and kept at 4°C. On the day of experiment, each specimen was mounted onto a torsion testing machine (Bionix^®^ EM Torsion Test System, MTS, Eden Prairie, MN USA) and angularly displaced at a rate of 2 degrees per second. Angular displacement and torque were recorded for the duration of each test. Torsional stiffness was computed as the gradient of the most linear part of the torque versus angular displacement curve. Angular failure displacement and failure torque were determined as the yield point of the torque versus angular displacement curve.

### Quantitative PCR Analysis

Fracture calluses were snap frozen in liquid nitrogen and processed using a bead beater with tungsten beads for homogenization. RNA was extracted from the cell lysate using Qiagen RNeasy RNA extraction kit. Total RNA was reverse transcribed into cDNA first strand using the Superscript II kit as per manufacturer’s protocol. Target and endogenous control genes were amplified with validated primers. Reactions were performed in triplicate in a 96-well plate using OneStep Plus (Applied Biosystems) for 40 cycles. Differential expression was determined using the comparative Ct method.

### Statistical Analysis

Results were presented graphically as a scatter plot of the individual sample values and expressed as a mean ± standard deviation of the mean. This format of presentation provides both the sample size in each group, as well as the result of each individual mice in the experiment. Sample normality was assessed with the Kolmogorov-Smirnov (KS) test prior to analysis. Statistical significance between all parametric sample groups was determined by performing an unpaired t test with Welch’s correction at 5% statistical significance. Individual P values for each test was presented. All data was analyzed using GraphPad PRISM (version 8.4.2; San Diego, California, USA).

## Results

### Loss of Hematopoietic Wnt Secretion Results in Marginal Decrease in Skeletal Size, but No Gross Defects

Wnts are implicated in both endochondral ossification and intramembranous ossification during skeletal growth and development. To investigate the role of hematopoietic Wnts in fetal skeletal development, we bred *Porcn^flox^* mice (32, 35) with *Vav-Cre* mice (37). This allowed us to generate mice with tissue specific deletion of *Porcn* in hematopoietic lineage cells (*Vav-Cre/Porcn^Del^*) (**Figure 1**). PORCN is required for Wnt secretion and activity (33, 38, 39). Hematopoietic lineage cells in *Vav-Cre/Porcn^Del^* mice therefore cannot produce active Wnts. *Porcn^Del^* mice were confirmed by genotyping (**Figure 1**). Depletion of hematopoietic Wnts was well tolerated, with similar numbers of *Vav-Cre/Porcn^Del^* and wild-type pups surviving.

**Figure 3:**
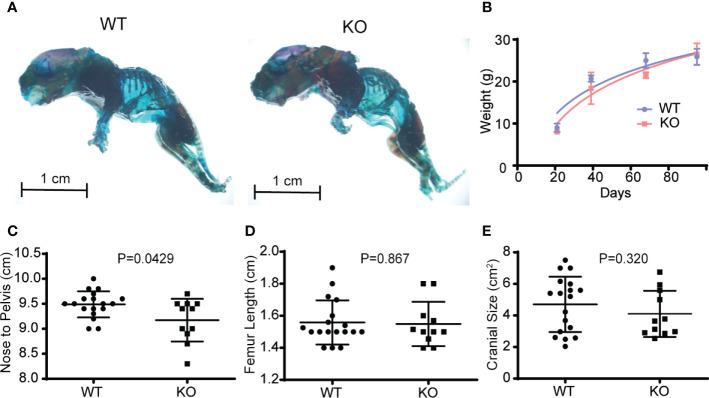
Depletion of hematopoietic Wnts has a minimal effect on musculoskeletal development. **(A)** Whole-mount skeletal staining demonstrating similar gross skeletal structure in the wild type (WT) and knockout mice (KO). **(B)** Growth curves demonstrated no growth arrest or delay in the KO mice. **(C)** Nose to pelvis length, **(D)** femur length, **(E)** cranial size (cross sectional dimensions) were comparable for the KO and WT mice, with only nose to pelvis length demonstrating a marginal increase in the WT.

If hematopoietic Wnts are important in fetal musculoskeletal development, we expect the *Vav-Cre/Porcn^Del^* pups to differ phenotypically from their wild-type littermates. The *Vav-Cre/Porcn^Del^* mice differed slightly in weight and size from the wild-type littermates but only at 3 months of age. They did not demonstrate any gross skeletal abnormalities on whole mount skeletal staining of the mice pups at 3 weeks of age (**Figure 3**). The microarchitecture of the spine was grossly normal on H&E and Safranin O staining, with normal development of the vertebral body and intervertebral discs, with no gross deformities. The *Vav-Cre/Porcn^Del^* mice pups also had normal weight gain over time until skeletal maturity and had normal long bone and cranial dimensions on maturation (**Figure 3**). There was a marginal decrease in the nose to pelvis length of *Vav-Cre/Porcn^Del^* mice but overall, there were no significant gross musculoskeletal abnormalities in these developing mice. There was no increase in mortality in the *Vav-Cre/Porcn^Del^* mice compared to their wild-type littermates, with similar numbers surviving beyond sexual maturity to late adulthood.

### Hematopoietic Wnts Do Not Contribute to Bone Mass Accrual

**Figure 4:**
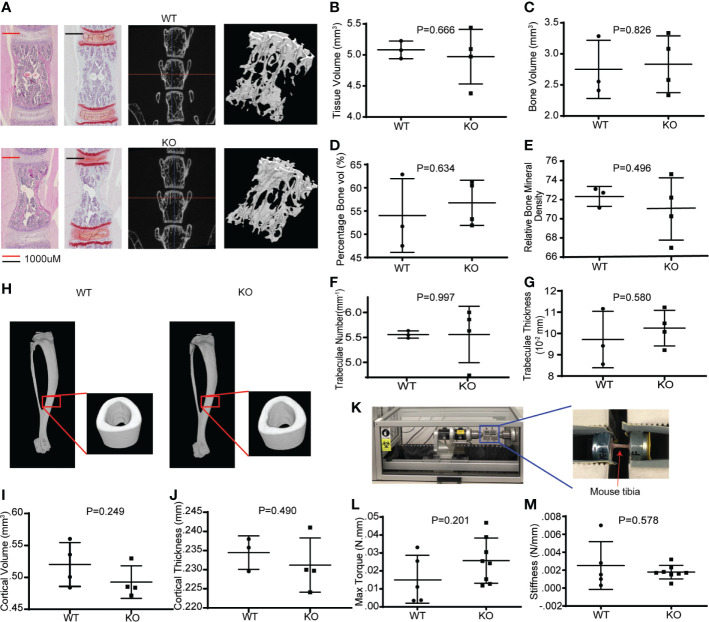
Hematopoietic Wnt-depleted mice have normal bone quality. **(A)** L5 vertebral bodies from WT and KO mice assessed by hematoxylin and eosin (H&E) and safranin O staining and micro computer-assisted tomography (μCT) with reconstruction demonstrating similar trabecular bone microarchitecture. **(B–G)** μCT quantification revealed no major differences in the bone quality of the L5 vertebrae by tissue volume (mm^3^), bone volume (mm^3^), percentage bone volume (%) and relative bone mineral density, trabaculae number(mm^-1^) and trabaculae thickness in the KO and WT mice. **(H)** μCT reconstruction of tibia cortical bone demonstrated minimal difference in **(I)** cortical volume (mm^3^), and **(J)** cortical thickness (mm) between the KO and WT mice. **(K)** Rotational torque machine. **(L)** The maximum tibia torque and **(M)** 4-point bending stiffness (Newton/mm) of the tibia were similar for both groups of mice.

Wnts are important in bone mass accrual and are implicated in osteoporosis. To determine if hematopoietic Wnts play an important role in bone mass accrual, we evaluated the microarchitecture of the *Vav-Cre/Porcn^Del^* vertebral body at maturity using high resolution microcomputer tomography scans (µCT) (**Figure 4**, male mice). The *Vav-Cre/Porcn^Del^* demonstrated no difference in trabeculae bone volume, percentage trabeculae bone volume and bone mineral density compared to the wild-type mice. There was also no difference in cortical volume and thickness. Since the dimensions of the cortical bone were similar, we proceeded to evaluate the qualitative properties of the cortical bone of the *Vav-Cre/Porcn^Del^* mice by measuring the stiffness and torque required for fracture (strength). If hematopoietic Wnts depletion resulted in quantitative or qualitative differences in bone development, we expect the cortical bone to have decreased mechanical strength. Again, there was no demonstrable difference in stiffness or torque strength between the *Vav-Cre/Porcn^Del^* mice and their wild-type littermates, suggesting that haemopoietic Wnts production is dispensable for physiological bone development.

### Hematopoietic Wnts Promote Fracture Callus Maturation by Up-Regulation of Osteoclasts

**Figure 5:**
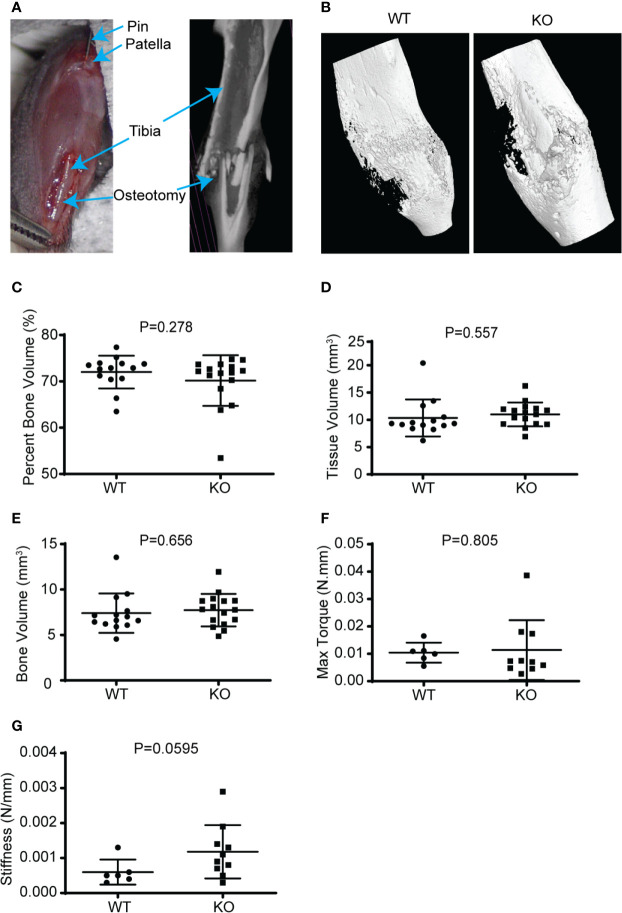
Hematopoietic depletion of Wnt does not affect callus volume and early healing. **(A)** Illustration of murine fracture model with a diaphyseal osteotomy stabilized with an intramedullary stainless-steel pin. **(B)** µCT reconstructed 3D images showed that the KO mice healing fracture callus had similar gross dimensions compared to the WT, 3 weeks after injury. **(C–E)** µCT volumetric analysis of the 3-week-old fracture callus by percentage bone volume (%), tissue volume (mm^3^), and bone volume (mm^3^); showed that the KO mice had normal callus bony volume and density. **(F, G)** Mechanical evaluation of the fracture callus at 3 weeks by maximum torque (Newton.mm), and stiffness (Newton/mm) were similar for both the KO and WT mice.

**Figure 6:**
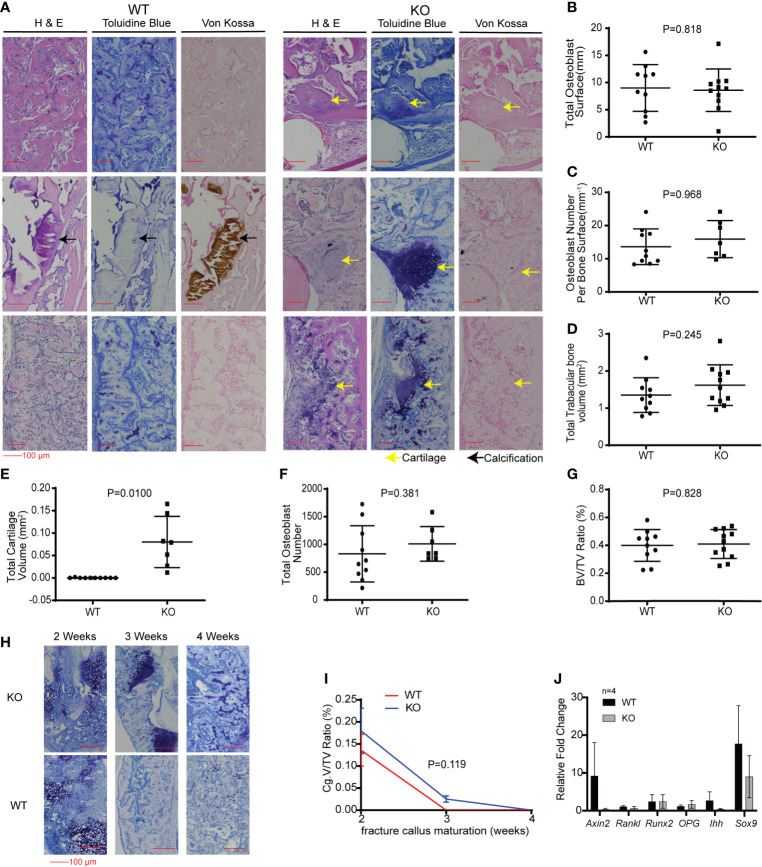
Hematopoietic Wnt-depleted mice had delayed callus maturation with more cartilage. **(A)** Histology of representative mice callus samples 3 weeks after injury from each group, demonstrated increased cartilage in the callus in the KO compared to the WT mice. **(B–H)** Quantitative evaluation of the fracture callus 3 weeks after injury were done by determining the **(B)** Total osteoblast surface (mm), **(C)** osteoblast number per bone surface (mm3), **(D)** total trabecular bone volume (mm3), **(E)** total cartilage volume (mm2), **(F)** total osteoblast number and **(G)** bone volume to total callus volume ratio (%). Total cartilage volume **(E)** was significantly less in the KO compared to the WT callus samples. **(H)** Representative images showing progressive maturation of the fracture callus in hematopoietic Wnt-depleted mice (KO) and wild type mice (WT). **(I)** Cartilage volume to total volume ratio (%) during the 1st 4 weeks after injury, demonstrating that the fracture calluses from the KO mice matured to woven bone after 4 weeks. **(J)** Quantitative polymerase chain reaction (qPCR) evaluation of the fracture callus at 3 weeks for relative expression of Wnt target gene (*Axin2*), bone turnover (*RankL*), osteoblastic genes (*Runx2, Opg*) and chondrogenic genes (*Ihh, Sox9*) revealed no significant difference in the 2 groups of mice. N=4 mice per group.

Fracture healing in long bones most frequently occurs by endochondral ossification. Direct healing only occurs when there is bone to bone apposition and compression at the fracture site, with absolute stability (40). The early phase of endochondral fracture healing involves an inflammatory process followed by the formation of a soft callus (41). This soft callus depends on chondrogenic differentiation of stromal mesenchymal stem cells, which then produce a cartilage scaffold around the fracture site. Specific Wnts (WNT9A, WNT5B) are known to play a key role in chondrocyte differentiation and soft callus formation (10). To investigate the role of hematopoietic Wnts in this early phase of fracture healing, we created an osteotomy in the tibia diaphysis of the *Vav-Cre/Porcn^Del^* mice, stabilized with an intra-medullary steel pin (**Figure 5**). We then evaluated the early fracture callus using high resolution micro-CT scans and histomorphometrically analysis at 2 weeks after the osteotomy had been performed. The hematopoietic Wnt depleted calluses were of normal volume and percentage bone volume compared to the wild-type calluses. There was only a marginal increase in the volume of cartilage, as well as the cartilage to total volume ratio in the hematopoietic Wnt-depleted callus (**Figure 6I**), indicating that the callus was less mature. However, this difference did not reach statistical significance. These results suggest that hematopoietic Wnts, specifically those implicated in chondrogenic differentiation, do not play an important role in the early phase of fracture healing.

**Figure 7:**
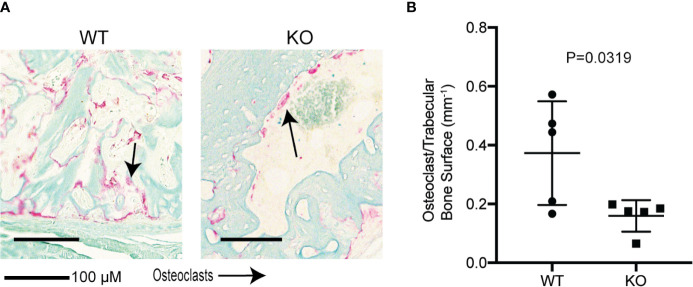
Hematopoietic Wnt depletion results in decreased osteoclasts in the fracture callus. **(A)** Representative histology (Tartrate resistant acid phosphatase staining) of fracture callus at 3 weeks showed fewer osteoclasts in the KO mice. **(B)** Osteoclast/trabecular bone surface of 3-week-old fractures were decreased in the KO mice.

We then investigated the effects of hematopoietic Wnt depletion in the 3-week-old callus. This allowed us to investigate the role of hematopoietic Wnts in the mineralization of the soft callus by osteoblasts. As expected, the wild-type callus demonstrated decreased cartilage composition, and increased woven bone and mineralization, compared to the 2-week-old callus. The *Vav-Cre/Porcn^Del^* mice also demonstrated a similar increase in woven bone and mineralization in the 3-week-old callus compared to the 2-week-old callus. Interestingly, we found that the *Vav-Cre/Porcn^Del^* mice had a higher volume of cartilage and percentage of cartilage in the callus at 3 weeks compared to the wild-type mice (**Figure 6**). The wild-type mice callus had negligible cartilage at 3 weeks after fracture, indicating that the callus had almost completely matured to woven bone. This suggested that hematopoietic Wnts had a positive effect in ossification of the cartilaginous callus. However, by 4 weeks, both the wild-type mice and the *Vav-Cre/Porcn^Del^* mice had fully matured calluses with all woven bone and no more cartilage. Thus, hematopoietic Wnt depletion demonstrated a clinically significant but dispensable role in callus formation and maturation. To identify how hematopoietic Wnt depletion resulted in a delay in callus remodeling to woven bone, we analyzed the osteoclasts and osteoblasts per bone surface area in the fracture calluses. We observed that the hematopoietic Wnt depleted callus had fewer osteoclasts at the 3-week mark (**Figure 7**). In contrast, the osteoblasts were not decreased.

We lysed the 3-week-old fracture callus to investigate the transcriptional changes in Wnt target genes and markers of osteoblastic differentiation (**Figure 6J**, female mice). Despite the difference in the phenotype of the callus at 3 weeks, expression of the Wnt/β-catenin target gene (*Axin2*) and the markers of osteoblastic differentiation (*Opg, Runx2*), bone resorption (*Rankl*) and early chondrogenesis (*Ihh* and *Sox9a*) were similar for both groups. This may be because the increase in Wnts secreted, and expression of the markers analyzed may have changed for only a short transient duration. This may have normalized at the time point of our analysis. There may also only be a difference in the ratio of specific Wnts rather than just a decrease in the quantity of β-catenin dependent Wnts in the KO callus compared to the wild-type callus.

## Discussion

This study sets out to investigate the role of hematopoietic Wnts in bone homeostasis and fracture healing. We know that bone marrow is important for hematopoiesis, but are hematopoietic cells important for bone formation? Do marrow cells play a role under normal physiological conditions in development or only in injury? These important questions can help us to understand how autologous bone marrow grafts assist in fracture healing. Stimulation of fracture healing by bone marrow or bone grafting has thus far been focused on the therapeutic effect of mesenchymal stem cells and the osteoconductive scaffold that the graft provides. We do not know much about how the hematopoietic component of the marrow contributes to bone formation. Understanding how hematopoietic cells modulate fracture healing will also help us to predict how bone marrow suppression or failure may affect fracture healing.

In this study, we found that hematopoietic Wnts had only a marginal quantitative effect on musculoskeletal development during fetal development and post-partum growth. However, when we examined what happens during fracture, we identified that there was a delay in fracture callus maturation. This was demonstrated by a more cartilaginous callus matrix. Fracture callus maturation is depended on two sequential and overlapping processes. The first process is resorption of the mineralized cartilage in the soft callus by osteoclasts. The second process is the replacement of the resorbed cartilage with woven bone by osteoblasts. Disruption of either of these two processes can result in delay in callus maturation (40). We found that in the hematopoietic Wnt depleted mice callus, there was a significant decrease in the osteoclast to bone surface area. This phenotype of delayed callus remodeling that we observed was similar to what Lin et al. (42) reported following reduction of osteoclast numbers in a fracture model. Can a shortfall in Wnts result in a reduced number of osteoclasts? Wei et al. (43) demonstrated that Wnt inhibition in osteoclast lineage cells by β-catenin deletion resulted in inhibition of proliferation of osteoclast precursor cells. Wnt/β-catenin activation stimulates GATA2/Evi1 expression which is required to generate osteoclast precursors. Constitutive Wnt/β-catenin activation resulted in proliferation of osteoclast precursors, but inhibition of osteoclastic differentiation to mature osteoclasts. Wei also showed that Wnt/β-catenin downregulation is needed for c-Jun activation, which in turn is required for the proliferation to differentiation switch in osteoclast precursors. This suggests that a very finely balanced, phased Wnt activation level is required for optimizing the number of mature osteoclasts. Although Wei’s experiments were not done in a fracture model, it follows that an acute shortfall in Wnt molecules during healing caused by hematopoietic Wnt depletion can disrupt this balance and result in fewer mature osteoclasts within the callus, specifically by inhibiting osteoclast precursor proliferation.

In contrast to the number of mature osteoclasts, the number of osteoblasts per bone volume was not decreased in the hematopoietic Wnt depleted callus. Osteoblasts play a key role in laying down bone during fracture healing, an essential step in callus maturation. Wnt signaling is a positive regulator of osteoblasts (44) and osteoblastic Wnt/β-catenin inhibition results in fracture non-union (14). In our model, Wnt secretion from osteoblasts was not blocked, and so the osteoblasts could provide an alternative source of functional Wnts in our model.

Other hematopoietic cells, specifically myeloid cells including macrophages and osteoclasts, have also been reported to be important for late stage fracture healing. Schlundt (41) reported that macrophage reduction did not result in an obvious effect in the early phase of fracture healing but resulted in delayed hard callus formation. They also reported that the anti-inflammatory M2 macrophage enhancement improved fracture callus maturation. They concluded that the macrophages played an important role in immune modulation during healing. Linda et al. (19) also reported that macrophages were important in promoting osteoblastic differentiation during fracture healing. Our study suggests that hematopoietic cells may contribute to fracture healing in a multitude of ways, including secretion of Wnts to increase osteoclasts during callus remodeling. This has important implications for our understanding of basic fracture healing physiology.

## Data Availability Statement

The raw data supporting the conclusions of this article will be made available by the authors, without undue reservation.

## Ethics Statement

The animal study was reviewed and approved by Institutional Animal Care and Use Committee.

## Author Contributions

Conception and design: KC and DV. Development of methodology: KC. Acquisition of data: KC, CC, AY, and EY. Analysis and interpretation of data: KC, VL, and DV. Writing, review, and/or revision of the paper: KC and DV. Administrative, technical, or material support: VL. Study supervision: KC and DV. All authors contributed to the article and approved the submitted version.

## Funding

This work was by the National Research Foundation Singapore, administered by the Singapore Ministry of Health’s National Medical Research Council under Singapore Translational Research (STaR) Award MOH-000155 to DV.

## Conflict of Interest

The authors declare that the research was conducted in the absence of any commercial or financial relationships that could be construed as a potential conflict of interest.
